# New Appliances & Things Medical

**Published:** 1910-05-14

**Authors:** 


### NEW APPLIANCES & THINGS MEDICAL.

[We shall be glad to receive at our Office, 28 & 29 Southampton Street, Strand, London, W.C., from the manufacturers, specimens of all new preparations and appliances.]

### HUGHES' PATENT SYRINGE.

(Messrs. May Roberts & Co., Ltd., London and Dublin.)

We have received from Mr. Gibbard Hughes, of Grocers' Hall Court, E.C., two specimens of a new patent syringe so constructed that any fluid which may have been forced behind the piston by the pressure of the downward or ejecting stroke is passed to the front again. This is effected by means of two parallel fluted channels in the glass at the nozzle end of the syringe, a simple and efficient mechanism which in practice works admirably. By this arrangement the escape of fluid at the mouth or handle end of the syringe during the upward or filling stroke is entirely avoided. We have used the syringes supplied, and find them in every way efficient, clean, and reliable. They are made of hard glass, in five sizes, holding respectively ¼, ½, 1, 1½, and 2 ounces, while larger sizes can be made to order and are fitted with either cotton or rubber pistons, or (as in the samples before us) with cotton-thread pistons. For syringing ears or any vertical work, in fact, these syringes are admirably suited, and appear to be a great advance on the old models. The wholesale agents are Messrs. May Roberts & Co., Ltd., of London and Dublin.

### STONWOOD FLOORING.

The floors of the new Nurses' Home of the Leicester Infirmary are well worth inspection by everyone who is interested in the burning question, "What is the best material for flooring?" They are examples of Stonwood Flooring. The floor is laid in two layers, each layer being three-eighths of an inch thick. The first layer is of special cork composition, on top of which the Stonwood proper is laid down. About 1,750 superficial yards were laid down in the Home, and the floors were finished up to the walls with a small coved skirting, of which 3,400 feet run had to be laid. This skirting prevents all accumulation of dust. The floors, which are as nearly perfect as it is possible to get them, being jointless, while the coved skirting prevents the harbouring of dust or septic material, are laid in two colours, red and yellow. They are easily cleaned, and are practically noiseless, while they feel as resilient and easy to the feet as do ordinary wood floors.

THE HOSPITAL. May 14, 1910.

